# Mosquitoes as vector-based sentinels for tracking bacterial pathogens and antimicrobial resistance in the environment

**DOI:** 10.3389/fmicb.2026.1925851

**Published:** 2026-08-11

**Authors:** Shicheng Chen

**Affiliations:** Medical Laboratory Sciences, College of Health and Human Sciences, Northern Illinois University, DeKalb, IL, United States

**Keywords:** antimicrobial resistance, bacterial pathogens, biosurveillance, mosquito microbiome, one health

## Abstract

Mosquitoes are increasingly recognized as mobile interfaces linking humans, animals, and diverse aquatic and terrestrial microbiomes. Beyond their established role as disease vectors, accumulating evidence indicates that mosquitoes harbor diverse bacterial communities and can acquire and potentially disseminate bacterial pathogens and antimicrobial resistance (AMR) determinants through feeding and environmental contact. Their dispersal capacity (typically 5–10 km, with occasional long-range wind-assisted movement), combined with a dual feeding ecology—blood feeding in females and nectar feeding in males—facilitates broad microbial exchange across ecosystems. These characteristics position mosquitoes as underutilized environmental sentinels for tracking microbial contamination and AMR dissemination. This mini-review highlights the potential of mosquitoes for detecting bacterial pathogens and AMR genes and emphasizes their emerging role within integrated One Health surveillance frameworks.

## 1 Introduction

Mosquitoes are among the most important arthropod vectors of human disease, responsible for substantial global morbidity and mortality, particularly in tropical and subtropical regions (Dahmana and Mediannikov, 2020). Conventional surveillance has primarily focused on protozoan parasites (especially for malaria and filarial nematodes), various viral pathogens (e.g., West Nile viruses, Dengue virus, Chikungunya virus and many others), vector geographic distribution, and insecticide resistance (Ravi et al., 2022; Wei et al., 2026). However, these approaches largely overlook the broader microbial ecosystems associated with mosquitoes, in part due to historically limited understanding of bacterial pathogens and their ecological roles (Nichols and Coon, 2025). The advent of next-generation sequencing has since reframed mosquitoes as holobionts, in which diverse bacterial communities influence vector competence, physiology, and pathogen transmission dynamics (Foo et al., 2024).

AMR has become a major threat to global public health, compromising the effective treatment of bacterial infections and undermining many modern medical interventions (Murray et al., 2022). In 2019, AMR was directly responsible for an estimated 1.27 million deaths and contributed to nearly 5 million deaths worldwide, with annual mortality projected to reach 10 million by 2050 if current trends continue (Murray et al., 2022; Naghavi et al., 2024). Despite increasing recognition of its ecological complexity, effective AMR surveillance remains challenging because resistant bacteria and antimicrobial resistance genes move continuously among clinical, agricultural, animal, and environmental settings, while existing surveillance systems are often compartmentalized and predominantly centered on human healthcare. Consequently, there is growing interest in complementary surveillance approaches capable of integrating microbial signals across interconnected ecosystems.

Mosquitoes undergo complete metamorphosis through egg, larval, pupal, and adult stages, typically completing development within 7–14 days under favorable conditions (Clements, 2023). Larvae develop in a wide range of aquatic habitats, from natural wetlands to anthropogenically influenced environments such as stormwater systems, wastewater, and agricultural runoff (Merritt et al., 1992; Skiff and Yee, 2014; Wang et al., 2024). These breeding sites are shaped by physicochemical conditions and human activities, which often serve as reservoirs of antimicrobial-resistant bacteria (ARB), and antimicrobial-resistance genes (ARGs) (Nichols and Coon, 2025; Larsson and Flach, 2022). As filter feeders, mosquito larvae continuously ingest environmental microorganisms, establishing gut microbial communities under strong ecological selection (Hegde et al., 2018). Microbial acquisition continues after emergence, as adults encounter additional microorganisms through nectar feeding, contact with vegetation, soil, and built environments, and blood feeding on vertebrate hosts, including livestock that may harbor AMR pathogens (Foster, 1995; Coon et al., 2014; Skiff and Yee, 2014; Clements, 2023). Combined with their ability to disperse typically 5–10 km from breeding sites, and occasionally much farther via wind-assisted movement (Verdonschot and Besse-Lototskaya, 2014), these ecological characteristics enable mosquitoes to integrate microbial signals from aquatic, terrestrial, and vertebrate-associated environments, supporting their potential as mobile biosentinels for monitoring bacterial pathogens and antimicrobial resistance within a One Health framework (Doyle et al., 2025).

## 2 Mosquitoes as reservoirs and sentinels for bacterial pathogens and antimicrobial resistance genes

### 2.1 Environmental acquisition and succession of the mosquito microbiome

**Figure 1:**
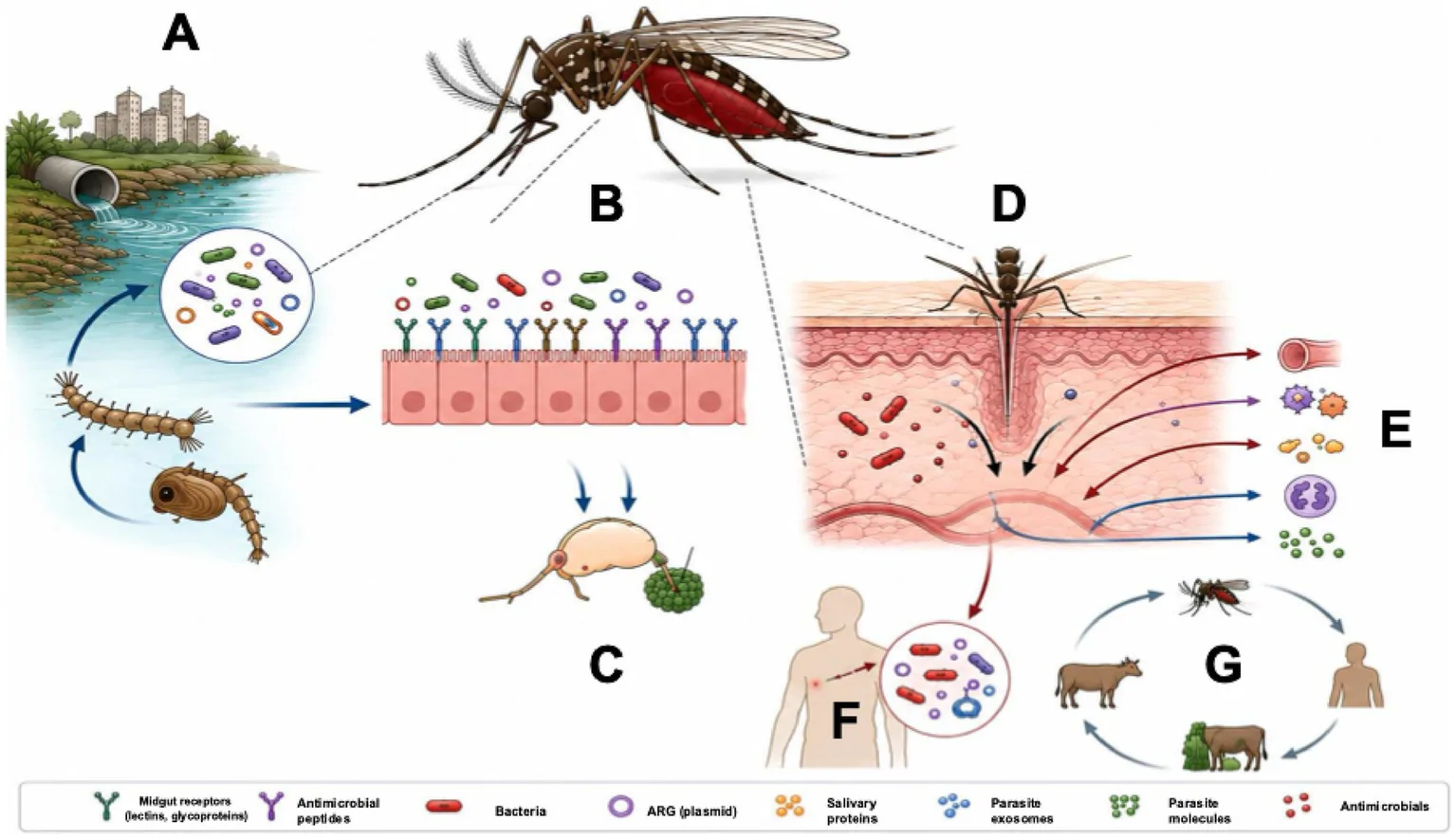
The role of mosquitoes in the acquisition, maintenance, and transmission of ARB and ARG. **(A)** Mosquitoes acquire susceptible bacteria, ARB, and ARGs from contaminated water environments during their aquatic larval stage; **(B)** ARB and ARGs on the adult mosquito surface and within the gut; **(C)** ARB survive mosquito immune responses, proliferate within the midgut, and disseminate to secondary tissues such as the hemocoel and salivary glands; **(D)** During a subsequent blood meal, infected mosquitoes co-inject egested microbiota containing ARB and ARGs into the vertebrate host; **(E)** Mosquito-derived factors and egested microbiota alter host hemostatic and innate immune responses. These changes enhance the survival and establishment of pathogens and ARB at the bite site; **(F)** Enhanced survival of pathogens and ARB within the skin promotes infection. ARB may colonize the host microbiota or contribute directly to disease development; **(G)** ARB and ARGs spread within the infected host, can be transmitted among humans and animals, and are reintroduced into the environment through waste and contaminated habitats, perpetuating the AMR cycle.

Microbial acquisition begins immediately after egg hatching. Larvae are indiscriminate filter feeders that consume suspended particles, bacteria, algae, and fungi present in aquatic breeding habitats (Grimstad and Walker, 1991). Consequently, breeding water serves as the primary reservoir for initial gut colonization (Figure 1A). Bacterial genera such as *Pseudomonas*, *Elizabethkingia*, Enterobacteriaceae members (e.g., *Enterobacter*, *Asaia* and *Serratia*) can be readily ingested and proliferate in the mosquito midgut before being shed in large numbers through feces (Chen et al., 2015, 2016, 2017, 2021). The transition from larva to pupa represents a major ecological bottleneck for microbial persistence (Galeano-Castañeda et al., 2020). During metamorphosis, larval tissues are extensively remodeled, and the digestive tract undergoes histolysis and regeneration. Most larval bacteria are eliminated during this process, resulting in a marked reduction in microbial biomass (Romoli et al., 2021).

However, several bacterial taxa persist through transstadial transmission, enabling recolonization of newly emerged adults (Figure 1B) (Galeano-Castañeda et al., 2020; Romoli et al., 2021). The efficiency of transstadial transmission varies considerably among bacterial species, with *Elizabethkingia*, *Asaia*, *Serratia*, and *Pseudomonas* frequently reported to survive developmental transitions (Minard et al., 2013a; Guégan et al., 2018). Following adult emergence, mosquitoes rapidly acquire additional microorganisms from diverse terrestrial environments. Sugar feeding introduces bacteria associated with floral nectar, fruits, plant surfaces, and environmental dust (Foster, 1995). Female mosquitoes instead ingest vertebrate-associated microorganisms during blood feeding, while mating, oviposition, and resting behaviors provide additional opportunities for microbial exchange (Sharakhov and Sharakhova, 2015). Consequently, adult mosquito microbiomes represent cumulative signatures of both aquatic larval habitats and terrestrial adult activities and may interact with or evade host immune defenses to establish in the midgut and exchange genetic material. Subsequently, microbes can disseminate to secondary tissues, including the hemocoel and salivary glands (Figure 1C).

Despite considerable geographic, ecological, and host-specific variation, mosquito-associated microbiomes are consistently enriched with a recurrent group of bacterial genera, including *Serratia*, *Elizabethkingia*, *Enterobacter*, *Pseudomonas*, *Acinetobacter*, and *Aeromonas* (Rodpai et al., 2023; Tong et al., 2023; Foo et al., 2024). These taxa are widely distributed across *Anopheles*, *Aedes*, and *Culex* mosquitoes (Thompson et al., 2015). They are frequently described as components of the mosquito “core microbiota” while no universal definition of a core mosquito microbiome has been established (Wang et al., 2011; Martinson and Strand, 2021). Unlike obligate insect symbionts that are transmitted vertically between generations, most mosquito-associated microorganisms originate from environmental sources (Coon et al., 2014). Collectively, these factors generate highly plastic and context-dependent microbiomes that reflect both mosquito biology and surrounding ecosystems (Guégan et al., 2018; Wang et al., 2024).

### 2.2 Ecological and host drivers of mosquito-associated microbiome dynamics

Mosquito gut microbiomes are highly dynamic and are shaped by interacting host, environmental, dietary, chemical, and mosquito species effects (Coon et al., 2014). Hosts strongly influence community structure through physiological traits such as gut pH, immune activity, and feeding behavior, resulting in species-specific microbiomes (Figures 1C–G) (Merritt et al., 1992). Environmental conditions, particularly larval breeding habitats (Walker et al., 1991; Merritt et al., 1992), further influence microbial acquisition, with water chemistry, nutrient availability, and pollution shaping the microbial communities available for colonization (Chen et al., 2026).

Anthropogenic activities, including wastewater discharge from hospitals and industries and agricultural runoff, often enrich breeding habitats with opportunistic and antibiotic-resistant bacteria (Berendonk et al., 2015), further supporting mosquitoes as indicators of environmental contamination. Temperature and seasonal variation introduce additional temporal changes by influencing both microbial growth and mosquito immune responses (Wang et al., 2011; Liu et al., 2024). Dietary shifts, particularly blood feeding, rapidly restructure gut communities through changes in nutrient availability, oxidative stress, and immune activation (Muturi et al., 2021). Chemical exposures, including insecticides, antibiotics, and heavy metals (Liu et al., 2024), further select for resistant bacterial taxa and promote enrichment of antimicrobial resistance genes (Alem et al., 2025). In addition, microbial interactions within the gut, including competition, quorum sensing, and bacteriocin production, regulate bacterial colonization and community stability (Steven et al., 2021; Giraud et al., 2022; Bogri et al., 2024). Collectively, these factors generate highly plastic and context-dependent microbiomes that reflect both mosquito biology and surrounding ecosystems. Although this variability complicates ecological interpretation, it also positions mosquitoes as valuable biological samplers of environmental microbial diversity (Giraud et al., 2022), antimicrobial resistance, and ecosystem disturbance, supporting their use in real-time surveillance of pathogen emergence and environmental change (Bogri et al., 2024).

### 2.3 Mosquito-associated clinically relevant bacteria and their implications for environmental sentinel surveillance

The frequent detection of clinically important bacterial taxa supports the use of mosquitoes as environmental sentinels for monitoring the distribution of opportunistic pathogens and AMR within a One Health surveillance framework (Gao et al., 2020). Core mosquito-associated taxa include *Elizabethkingia anophelis*, *Serratia marcescens*, members of the *Enterobacter cloacae* complex, *Klebsiella pneumoniae*, *Pseudomonas aeruginosa*, *Acinetobacter baumannii*, and *Aeromonas hydrophila,* all of which are recognized opportunistic human pathogens capable of causing bacteremia, pneumonia, urinary tract infections, wound infections, and meningitis (Table 1) (Minard et al., 2013a; Guégan et al., 2018; Gao et al., 2020; Liu et al., 2024). Experimental studies have demonstrated that mosquito-derived *Elizabethkingia*, *Serratia*, and *Asaia* isolates successfully recolonize, proliferate, and persist following reintroduction into multiple mosquito species, supporting their stable association with mosquito hosts and persistence across vector populations, although evidence for clinical transmission remains limited (Chen et al., 2014, 2015, 2017, 2021, 2024; Howard et al., 2020; Hem et al., 2022). In addition to these recurrent mosquito-associated bacteria, several multidrug-resistant opportunistic pathogens, including methicillin-resistant *Staphylococcus aureus* (Arif et al., 2024), *Escherichia–Shigella* group (Muturi et al., 2017), and *Stenotrophomonas maltophilia* (Hughes et al., 2016), have also been isolated from mosquitoes. Although these organisms are generally considered transient or environmentally acquired rather than stable members of the mosquito microbiota, their repeated detection suggests frequent exposure of mosquitoes to wastewater, fecally contaminated environments, and other anthropogenically influenced aquatic habitats.

**Table 1:** Representative antimicrobial-resistant bacteria identified from mosquitoes.

Core microbiota	Mosquito hosts	Pathogenesis potentials in humans	Known AMR	Detection methods	References
*Elizabethkingia anophelis*	*Anopheles, Aedes and Culex*	Various diseases such as septicemia	*β*-lactams (including carbapenems), aminoglycosides, macrolides, tetracyclines, and fluoroquinolones	Culture, WGS, and metagenomics	Onyango et al. (2021), Mosquera et al. (2023), Villegas et al. (2023), Chen et al. (2024)
*Serratia marcescens*	*Anopheles, Aedes and Culex*	Various diseases such as septicemia, UTI, wounds	β-lactam, aminoglycosides, macrolides, tetracyclines and glycopeptides	Culture, WGS and metagenomics	Chen et al. (2017), Heu et al. (2021), Tavares-Carreon et al. (2023), Barnes et al. (2025)
*E. cloacae* complex	*Anopheles*, *Aedes*	Various diseases such as septicemia, UTI, respiratory infections, Intra-abdominal infections	β-lactams (AmpC), extended-spectrum cephalosporins, carbapenems, aminoglycosides, fluoroquinolones	Culture, WGS and metagenomics	Ezemuoka et al. (2020), Díaz et al. (2025), Macesic et al. (2025)
*Klebsiella pneumoniae*	*Aedes aegypti* *Aedes albopictus*	Various diseases such as septicemia, UTI, respiratory infections, Intra-abdominal infections	β-lactams (ESBLs), aminoglycosides, fluoroquinolones, tetracyclines, polymyxins	Culture and metagenomics	Mosquera et al. (2021), Villegas et al. (2023), Sambanthan et al. (2026)
*Acinetobacter baumannii*	*Aedes albopictus*	Opportunistic infections including pneumonia, bacteremia, UTI, wound infections (ESKAPE pathogen)	β-lactams (including carbapenems), aminoglycosides, fluoroquinolones, tetracyclines, polymyxins	Culture and metagenomics	Minard et al. (2013b)
*Pseudomonas aeruginosa*	*Anopheles gambiae*, *Aedes aegypti*, *Aedes albopictus*, *Culex quinquefasciatus*	Opportunistic infections including septicemia, pneumonia, urinary tract infections, and wound infections	β-lactams, carbapenems, aminoglycosides, fluoroquinolones, polymyxins	Culture, WGS, metagenomics, MALDI-TOF MS	Pidiyar et al. (2004), Saab et al. (2020), Singh et al. (2022)
*Aeromonas hydrophila*	*Culex pipiens*, *Aedes albopictus, Aedes aegypti*	Opportunistic gastroenteritis, wound infections	β-lactams, tetracyclines, fluoroquinolones, sulfonamides	Culture, experimental infection, WGS	Rodpai et al. (2023), Wehbe et al. (2025)

Beyond taxonomic composition, recent genome-resolved investigations have substantially strengthened the evidence supporting mosquitoes as reservoirs of clinically relevant AMR determinants (Table 1). Large-scale sequencing efforts, including the MosAIC project, have generated extensive genome collections from mosquito-associated bacteria, enabling direct comparisons with clinical isolates (Foo et al., 2024). These comparative genomic analyses demonstrate that mosquito-derived pathogens retain the same major AMR determinants identified in human infections. For example, mosquito-associated *E. anophelis* consistently harbors the conserved chromosomal *β*-lactamase genes *blaB*, *blaGOB*, and *blaCME*, which are the hallmark resistance determinants of clinical *E. anophelis* isolates and confer intrinsic resistance to numerous β-lactam antibiotics, including carbapenems (Chen et al., 2024). Similarly, mosquito-associated *S. marcescens*, members of the *E. cloacae* complex, *K. pneumoniae*, *A. baumannii*, and *P. aeruginosa* possess genomic features characteristic of their clinically important counterparts, including AmpC β-lactamases, multidrug efflux systems, extended-spectrum β-lactamases, carbapenemases, and other acquired resistance determinants (Macesic et al., 2025). These findings indicate that mosquitoes harbor bacterial populations with AMR gene repertoires comparable to those recovered from human infections, reinforcing their value as mobile biosentinels for monitoring the environmental emergence and dissemination of clinically relevant antimicrobial resistance within a One Health framework.

Although mosquitoes are not recognized as biological vectors for most bacterial pathogens, experimental studies have demonstrated that certain bacteria can survive within mosquitoes and may exhibit transmission potential under specific experimental conditions (Akhouayri et al., 2013; Muturi et al., 2021; Accoti et al., 2023). For example, *Rickettsia felis*, the causative agent of flea-borne spotted fever, has also been detected in wild-caught mosquitoes (Mediannikov et al., 2013; Dieme et al., 2015). Mosquitoes carrying the bacterium were permitted to feed on mice, and the bacterium was subsequently detected in the blood of the exposed hosts. This pattern is consistent with a potential role for saliva-mediated transfer during feeding, although the broader ecological and epidemiological significance remains uncertain, suggesting that mosquito–bacterium interactions may be more complex than previously appreciated (Dieme et al., 2015). Another study demonstrated that viable *S. marcescens* isolated from mosquito saliva could be introduced into a mammalian host during blood feeding (Accoti et al., 2023). These findings indicate that mosquitoes can harbor bacteria and, under specific circumstances, transfer them during blood feeding. However, such observations remain limited to a small number of bacterial species and largely originate from controlled laboratory experiments rather than natural transmission cycles (Mediannikov et al., 2013; Dieme et al., 2015).

It is well known that the insect gut provides a favorable microenvironment for bacterial persistence and horizontal gene transfer (HGT), making it a potential ecological niche for the maintenance and dissemination of AMR (Gwenzi et al., 2021; Vieira-Alcântara et al., 2025). Following ingestion, bacteria from diverse environmental and host-associated sources are concentrated within the gut lumen, where high cell densities, close physical proximity, and biofilm formation promote interbacterial contact (Yin et al., 2022). The gut environment is further characterized by abundant nutrients, fluctuating oxygen gradients, variable pH, and host-derived antimicrobial peptides, all of which impose selective pressures that shape microbial community composition while maintaining metabolically active bacterial populations (Idigo et al., 2025). These conditions facilitate bacterial conjugation, transformation, and bacteriophage-mediated transduction, thereby increasing opportunities for the exchange of mobile genetic elements, including plasmids, integrons, and transposons (Odetoyin et al., 2020). Mosquito-associated bacteria frequently harbor mobile genetic elements (MGEs), including plasmids, integrons, and transposons, which facilitate HGT and contribute to bacterial adaptation and the dissemination of AMR determinants (Teo et al., 2014; Chen et al., 2024; Foo et al., 2024). Within the mosquito gut, high microbial density, fluctuating nutrient availability, and host immune modulation create conditions that may promote close interbacterial interactions and genetic exchange (Liu et al., 2024). Bacterial genera commonly associated with mosquitoes, including *Enterobacter*, *Pseudomonas*, and *Acinetobacter*, are well-established reservoirs of plasmid-mediated resistance determinants and have a demonstrated capacity for HGT in environmental and clinical settings (Rozwandowicz et al., 2018; Mei et al., 2024; Tobin et al., 2025).

*Elizabethkingia* species, as predominant and stably associated members of the mosquito microbiota, possess conjugative integrative elements capable of transferring between members of the Flavobacteriaceae, including *Chryseobacterium*, highlighting the potential for intergeneric dissemination of mobile resistance determinants (Fu et al., 2021). In addition, conjugative transposons carrying resistance genes have been experimentally transferred from the anaerobic commensal bacterium *Prevotella jejuni* into *Elizabethkingia* spp. (Yang et al., 2024), where they were stably maintained, demonstrating the capacity of *Elizabethkingia* to acquire resistance determinants through horizontal gene transfer and underscoring the importance of continued genomic surveillance. Mobile genetic elements also influence bacterial evolution beyond AMR. During the 2015–2016 Midwestern U.S. outbreak of *E. anophelis*, comparative genomic analyses identified outbreak-specific transposon insertions that were associated with enhanced adaptation and may have contributed to increased virulence besides AMR, illustrating how genome plasticity can facilitate the emergence of clinically significant lineages (Perrin et al., 2017; Hu et al., 2022). Once acquired, resistant bacteria and their associated resistance genes may be redistributed through mosquito excreta, oviposition, and carcass decomposition, thereby contributing to the movement of AMR determinants between aquatic and terrestrial environments (Hem et al., 2022).

### 2.4 Microbiome and antimicrobial resistance profiling methods

An important methodological consideration in mosquito microbiome surveillance is distinguishing the internal microbiome from microorganisms associated with the external body surface. The internal microbiome represents persistent host-associated communities, whereas cuticle-associated bacteria generally reflect recent environmental contamination acquired from breeding water, vegetation, soil, or vertebrate hosts (Guégan et al., 2018; Liu et al., 2024). Consequently, sampling strategies should match the study objective. Surface sterilization before tissue dissection or DNA extraction is commonly used to characterize the internal microbiome while minimizing external contamination. In contrast, whole-body nucleic acid extraction without surface sterilization preserves both internal and surface-associated microorganisms, providing a more comprehensive representation of recent environmental microbial exposure.

Mosquito-associated microbiomes have been widely investigated using a combination of culture-dependent (culturomics) and culture-independent approaches (Chen et al., 2014, 2021, 2024; Long et al., 2024). Culturomics have been used in parallel to isolate viable bacterial strains from mosquito samples (Chen et al., 2015, 2017, 2021, 2024). Isolates were obtained through standard cultivation techniques, followed by phenotypic characterization and antimicrobial susceptibility testing. Species-level identification was further confirmed using MALDI-TOF mass spectrometry (Tandina et al., 2016; Costa et al., 2024). Despite its advantages, culturomics is inherently limited by its dependence on microbial growth under laboratory conditions. Consequently, many strict anaerobes, fastidious organisms, and other unculturable bacteria cannot be isolated using conventional artificial media, resulting in an incomplete representation of the microbial community. However, pure cultures can be tested for their antimicrobial resistance profiles. Moreover, they were subjected to whole-genome sequencing (WGS) to enable high-resolution genomic characterization, including identification of AMR genes, virulence factors, and mobile genetic elements such as plasmids, integrons, transposons, and prophages (Foo et al., 2024).

Bacterial community structure is commonly profiled using 16S rRNA gene sequencing as a culture-independent method (Díaz et al., 2025). Briefly, genomic DNA was extracted from mosquito samples, and bacterial 16S rRNA gene fragments were amplified using universal primer sets targeting variable regions (commonly V3–V4 or V4). Amplicons were sequenced on a next-generation sequencing platform, and raw reads were processed using standard bioinformatic pipelines, including quality filtering, chimera removal, and amplicon sequence variant inference (Caporaso et al., 2012). Taxonomic assignment was performed against curated reference databases, and diversity metrics were used to assess microbial community composition and structure (Knight et al., 2018). Although 16S rRNA gene sequencing does not directly detect AMR genes, it provides essential taxonomic context for identifying potential bacterial hosts of resistance determinants and supports downstream integration with targeted qPCR/dPCR and metagenomic analyses (Al Amin et al., 2022; Kakooza et al., 2025).

Shotgun metagenomic sequencing was employed to profile the total microbial and functional gene content of mosquito-associated communities without the need for cultivation (Chandler et al., 2015; Thongsripong et al., 2021). Metagenomic reads were processed for taxonomic and functional annotation, including profiling of antimicrobial resistance genes (resistome), metabolic pathways, and genes associated with stress response, biofilm formation, motility, and host adaptation (Doster et al., 2020). This approach enabled quantification of AMR gene abundance and distribution across mosquito developmental stages, tissues, and environmental conditions. Integrative analyses combining 16S rRNA gene sequencing, culture-based isolation, WGS, and metagenomics were used to improve the resolution of microbial community structure and functional potential. This multi-tiered approach allowed discrimination between transient environmental bacteria and stable mosquito-associated taxa, as well as linkage of taxonomic identity with functional traits relevant to vector competence and antimicrobial resistance dissemination (Al Amin et al., 2022).

## 3 Discussion

Mosquitoes are increasingly recognized as more than vectors of arboviral and parasitic diseases; they also function as ecological interfaces linking environmental, animal, and human microbiomes (Dahmana and Mediannikov, 2020). Their aquatic larval habitats, diverse feeding behaviors, and mobility enable them to acquire microorganisms from environmental, animal, and human sources, positioning them as mobile interfaces that integrate microbial signals across multiple ecosystems (Merritt et al., 1992). Advances in culture-dependent methods, high-throughput sequencing, metagenomics, and comparative genomics have substantially expanded our understanding of mosquito-associated bacterial communities and demonstrated that mosquitoes frequently harbor clinically important opportunistic pathogens and AMR determinants (Minard et al., 2013a; Tandina et al., 2016). The growing availability of genome-resolved datasets, exemplified by the MosAIC project, further shows that mosquito-associated bacteria can possess genomic characteristics comparable to those of clinical isolates, supporting the use of mosquitoes as complementary biosentinels within One Health surveillance programs (Foo et al., 2024).

Despite these advances, several challenges remain. Distinguishing stable mosquito-associated microbiota from transient environmental contamination is essential for interpreting surveillance data and requires standardized sampling strategies and nucleic acid extraction protocols tailored to specific study objectives. Moreover, the detection of bacterial pathogens or AMR genes in mosquitoes does not necessarily indicate biological transmission, and their ecological and epidemiological significance should be interpreted alongside environmental, clinical, and veterinary surveillance data. Future studies should integrate longitudinal field sampling with culture-based isolation, whole-genome sequencing, metagenomics, and resistome analyses to better characterize the persistence, transmission dynamics, and public health relevance of mosquito-associated bacteria. Together, these multidisciplinary approaches will improve our understanding of microbial movement across environmental and host-associated interfaces and establish mosquitoes as valuable complementary tools for integrated One Health surveillance of bacterial pathogens and antimicrobial resistance.
